# Supplementary material for: Multilingual validation of the short form of the Unesp-Botucatu Feline Pain Scale (UFEPS-SF)
Source: PeerJ. 2022 Mar 23;10:e13134. doi: 10.7717/peerj.13134 (PMC8957279; doi:10.7717/peerj.13134)
Supplement: Table S1 [file peerj-10-13134-s001.docx]

**CHINESE – 中文**

**貓隻疼痛UFEPS-SF簡化評估表**

([www.animalpain.org](http://www.animalpain.org))

| **項目** | **臨床表現** | **評分** |
| --- | --- | --- |
| **評估貓隻在籠內所呈現的姿勢兩分鐘** | | |
| **1** | 姿態自然, 放鬆, 且/或是正常移動 | 0 |
|  | 姿態自然但緊張, 不移動、或小幅度移動、或拒絕移動 | 1 |
|  | 呈現拱背姿勢且/或是背側卧姿勢 | 2 |
|  | 頻繁改變姿勢或是焦躁不安 | 3 |
|  | | **勾選合適的項目** |
| **2** | 貓隻收縮又伸展後肢，且/或收縮其腹部肌肉 (側腹) |  |
|  | 貓隻眼睛呈現半閉 (需待麻醉結束一小時後始可將此項目列入評分) |  |
|  | 貓隻舔拭且/或是啃咬手術傷口 |  |
|  | 貓隻強烈搖擺尾巴 |  |
|  | *上述行為皆不存在* | 0 |
|  | *具有上述其中一項行為* | 1 |
|  | *具有上述其中兩項行為* | 2 |
|  | *具有上述其中三項或是全部行為* | 3 |
|  | | |
| **評估貓隻在籠子打開後的舒適度**，**活動力**，**精神狀態**，**以及貓隻對於與觀察者及/或周圍環的注意力** | | |
| **3** | 舒適並留意關心周圍環境 | 0 |
|  | 安靜並稍微留意周圍環境 | 1 |
|  | 安靜而且不關心周圍環境. 貓隻可能背對籠子出口 | 2 |
|  | 不舒適, 焦躁不安,可能稍微留意或是不關心周圍環境. 貓隻可能背對籠子出口 | 3 |
|  | | |
| **評估貓隻對於觸碰與按壓疼痛處的反應** | | |
| **4** | 無反應 | 0 |
|  | 對於觸碰疼痛處無反應,但對按壓有反應 | 1 |
|  | 觸碰與按壓疼痛處都有反應 | 2 |
|  | 不接受任何觸碰或是按壓 | 3 |

**FRENCH - FRANÇAIS**

**Formulaire abrégé de l’échelle de douleur feline de l’Unesp-Botucatu – UFEPS-SF**

([www.animalpain.org](http://www.animalpain.org))

| **ITEM** | **Description** | **Score** |
| --- | --- | --- |
| **Évaluation de la posture du chat dans la cage pendant 2 minutes** | | |
| **1** | Naturel, détendu et/ou bouge normalement | 0 |
|  | Naturel mais tendu, bouge peu, pas ou est réticent à bouger | 1 |
|  | Dos arqué et/ou en décubitus dorso-latéral | 2 |
|  | Change de position fréquemment ou est agité | 3 |
|  | | **Cocher la case qui s’applique** |
| **2** | Le chat contracte et étend ses membres pelviens et/ou contracte ses muscles abdominaux (flancs) |  |
|  | Les yeux du chat sont mi-clos (ne pas tenir compte de ce point dans l’heure suivant la fin de l’anesthésie) |  |
|  | Le chat lèche et/ou mord le site douloureux |  |
|  | Le chat bouge frénétiquement sa queue |  |
|  | *Aucun des comportements mentionnés ci-dessus n’est présent* | 0 |
|  | *Présence d’un des comportements parmi ceux mentionnés ci-dessus* | 1 |
|  | *Présence de deux des comportements parmi ceux mentionnés ci-dessus* | 2 |
|  | *Présence de trois ou de l’ensemble des comportements mentionnés ci-dessus* | 3 |
|  | | |
| **Évaluation du confort, de l’activité et de l’attitude du chat après ouverture de la cage et son attention envers l’observateur et/ou l’environnement** | | |
| **3** | Confortable et attentif | 0 |
|  | Tranquille et peu attentif | 1 |
|  | Tranquille et inattentif. Le chat peut faire face au fond de la cage | 2 |
|  | Inconfortable, agité et peu ou pas attentif. Le chat peut faire face au fond de la cage | 3 |
|  | | |
| **Évaluation de la réaction du chat au toucher suivi d’une pression autour du site douloureux** | | |
| **4** | Ne réagit pas | 0 |
|  | Ne réagit pas lorsque le site douloureux est touché, mais réagit lorsqu’il est palpé avec une pression légère | 1 |
|  | Réagit au toucher et à la pression du site douloureux | 2 |
|  | Ne se laisse pas palper | 3 |

**GERMAN - DEUTSCH**

**Kurzform der UNESP-Botucatu Schmerz-Skala für Katzen- UFEPS-SF**

([www.animalpain.org](http://www.animalpain.org))

| **ITEM** | **Description** | **Score** |
| --- | --- | --- |
| **Beobachten Sie die Körperhaltung der Katze im Käfig für 2 Minuten** | | |
| **1** | Unauffällig, entspannt, bewegt sich normal | 0 |
|  | Unauffällige Körperhaltung, jedoch leicht angespannt;  Bewegt sich nicht oder wenig oder zögerlich | 1 |
|  | Gekrümmte Körperhaltung und/oder dorso-laterale Liegeposition | 2 |
|  | Ändert häufig die (Sitz-) Position, unruhig | 3 |
|  | | |
| **2** | Die Katze spannt die Hintergliedmassen an und/oder streckt diese aus Zusätzlich oder auch alleinstehend kann ein Anspannen der abdominalen Muskeln in der Flanke beobachtet werden |  |
|  | Die Augen der Katze sind halb geschlossen (dieser Punkt wird bis zu einer Stunde nach Ende der Anästhesie NICHT bewertet) |  |
|  | Die Katze leckt und/oder beißt die schmerzende Stelle |  |
|  | Die Katze bewegt ihren Schwanz energisch hin und her |  |
|  | *Keine der oben genannten Verhaltensweisen sind vorhanden* | 0 |
|  | *Eine der oben genanntenVerhaltensweisen konnte beobachtet werden* | 1 |
|  | *Zwei der oben genannten Verhaltensweisen konnten beobachtet werden* | 2 |
|  | *Drei oder alle der oben genannten Verhaltenweisen konnten beobachtet werden* | 3 |
|  | | |
| **Nach Öffnung des Käfigs: Beurteilung von Wohlbefinden, Aktivität und Haltung der Katze sowie**  **Aufmerksamkeit der Katze der Umwelt (dem Beobachter und/oder der Umgebung) gegenüber** | | |
| **3** | Beschwerdefrei und aufmerksam/ interessiert an der Umwelt | 0 |
|  | Ruhig und wenig interessiert an der Umwelt | 1 |
|  | Ruhiges Verhalten, kein Interesse an der Umwelt, Katze sitzt unter Umständen mit dem Gesicht zur Rückwand des Käfigs da | 2 |
|  | Unzufrieden, unruhig, wenig oder nicht aufmerksam. Katze sitzt eventuell mit dem Kopf zur Rückseite des Käfigs | 3 |
|  | | |
| **Beurteilung der Reaktion der Katze bei Berührung, gefolgt von Palpation im Bereich der schmerzhaften Stelle** | | |
| **4** | Keine Reaktion | 0 |
|  | Keine Reaktion, wenn schmerzhafter Bereich leicht berührt wird, aber Reaktion bei leichtem Druck | 1 |
|  | Reaktion bei leichter Berührung und bei leichtem Druck | 2 |
|  | Lässt keine Berührung zu | 3 |

**ITALIAN - ITALIANO**

**Versione breve della scala di valutazione del dolore del gatto Unesp-Botucatu – UFEPS-SF**

([www.animalpain.org](http://www.animalpain.org))

| **ITEM** | **Descrizione** | **Punteggio** |
| --- | --- | --- |
| **Valutare per 2 minuti la postura del gatto all’interno della gabbia** | | |
| **1** | Naturale, rilassato e/o si muove normalmente | 0 |
|  | Naturale ma contratto, non si muove o si muove poco o è riluttante a muoversi | 1 |
|  | Posizione incurvata e/o decubito dorso-laterale | 2 |
|  | Cambia frequentemente posizione o è irrequieto | 3 |
|  | | **Spuntare l’indicatore se presente** |
| **2** | Il gatto contrae ed estende gli arti posteriori e/o contrae i muscoli addominali (fianco) |  |
|  | Gli occhi del gatto sono parzialmente chiusi (non considerare questo indicatore se presente fino ad un’ora dopo la fine dell’anestesia) |  |
|  | Il gatto lecca e/o morde la parte dolente |  |
|  | Il gatto muove la coda con forza |  |
|  | *Tutti i comportamenti anzidetti sono assenti* | 0 |
|  | *Presenza di uno dei comportamenti anzidetti* | 1 |
|  | *Presenza di due dei comportamenti anzidetti* | 2 |
|  | *Presenza di tre o tutti i comportamenti anzidetti* | 3 |
|  | | |
| **Valutazione del comfort, dell'attività e dell'atteggiamento del gatto all’apertura della gabbia, e del suo interesse nei confronti dell’osservatore e/o dell’ambiente circostante** | | |
| **3** | A suo agio ed interessato | 0 |
|  | Calmo e poco interessato | 1 |
|  | Calmo e non interessato. Il gatto può essere rivolto verso la parte posteriore della gabbia | 2 |
|  | A disagio, irrequieto, poco o per niente interessato. Il gatto può essere rivolto verso la parte posteriore della gabbia | 3 |
|  | | |
| **Valutazione della reazione del gatto quando toccato e poi delicatamente palpato intorno alla parte dolente** | | |
| **4** | Non reagisce | 0 |
|  | Non reagisce quando si tocca la parte dolente, ma reagisce alla palpazione delicata della parte | 1 |
|  | Reagisce sia quando la parte dolente viene toccata che quando viene palpata | 2 |
|  | Non permette la palpazione | 3 |

**JAPANESE – 日本語**

**簡略版UNESP-Botucatu 疼痛スケール　≪猫用≫**

([www.animalpain.org](http://www.animalpain.org))

| **項目** | **評価内容** | **スコア** |
| --- | --- | --- |
| **ケージ内の猫の様子を2分間観察評価する** | | |
| **1** | 自然体でリラックスしているまたは通常通りに動いている。 | 0 |
|  | 自然体であるが、緊張している。あまり動かないか動きたがらない。 | 1 |
|  | 背中を丸めてうずくまっているまたは横たわっている（側臥・背臥位） | 2 |
|  | 頻繁に体位を変えるまたは落ち着きがない。 | 3 |
|  | | **当てはまる項目をチェックする** |
| **2** | 後肢の伸縮または腹筋（側腹部）の緊張や収縮がみられる。 |  |
|  | 目を半ば閉じている |  |
|  | 術創を舐めたり、噛んでいる。 |  |
|  | 尾を強く振っている |  |
|  | *上記の行動は一つも見られない* | 0 |
|  | *上記の行動のうち一つがみられる* | 1 |
|  | *上記の行動のうち二つがみられる* | 2 |
|  | *上記の行動のうち三つもしくはすべてが見られる。* | 3 |
|  | | |
| **ケージを開けた後の猫の様子、活動と態度、および観察者と周囲の環境に対する反応を評価する** | | |
| **3** | 快適な様子で周囲に普通通り反応している | 0 |
|  | 静かで若干反応している | 1 |
|  | 静かで反応を示さない、またはこちらに背中を向けている。 | 2 |
|  | 居心地が悪そうで落ち着きがない。多少の反応があるか、全く反応しない。またはこちらに背中を向けている。 | 3 |
|  | | |
| **痛みのある患部周辺を触って軽く押した時の猫の反応を評価する** | | |
| **4** | 反応しない | 0 |
|  | 触るだけでは反応しないが、軽く押すと嫌がる | 1 |
|  | 触るだけでも嫌がる | 2 |
|  | 触ることが出来ない | 3 |

**PORTUGUESE - PORTUGUÊS**

**Versão curta da escala de dor em felinos da Unesp-Botucatu - UFEPS-SF**

([www.animalpain.org](http://www.animalpain.org))

| **ITEM** | **Descripción** | **Escores** |
| --- | --- | --- |
| **Avalie a postura do gato no gatil por 2 minutos** | | |
| **1** | Natural, relaxado e/ou se movimenta normalmente | 0 |
|  | Natural, mas tenso, não se movimenta ou se movimenta pouco ou está relutante em se mover | 1 |
|  | Postura arqueada e/ou decúbito dorso-lateral | 2 |
|  | Muda de posição frequentemente ou inquieto | 3 |
|  | | **Marque os itens que ocorrem** |
| **2** | O gato contrai e estende os membros pélvicos e/ou contrai os músculos abdominais (flanco) |  |
|  | Os olhos do gato estão parcialmente fechados (não considere este item caso presente até 1 h após o fim da anestesia) |  |
|  | O gato lambe e/ou morde a área afetada |  |
|  | O gato movimenta a cauda fortemente |  |
|  | *Todos os comportamentos acima estão ausentes* | 0 |
|  | *Presença de um dos comportamentos acima* | 1 |
|  | *Presença de dois dos comportamentos acima* | 2 |
|  | *Presença de três ou todos os comportamentos acima* | 3 |
|  | | |
| **Avalie o conforto, atividade e atitude após o gatil ser aberto e o quão atento o gato está ao observador e/ou ambiente** | | |
| **3** | Confortável e atento | 0 |
|  | Quieto e pouco atento | 1 |
|  | Quieto e não atento. O gato pode estar voltado para a parte de trás do gatil | 2 |
|  | Desconfortável, inquieto e pouco atento ou não atento. O gato pode estar voltado para a parte de trás do gatil | 3 |
|  | | |
| **Avalie a reação do gato ao toque, seguido de pressão ao redor do local dolorido** | | |
| **4** | Não reage | 0 |
|  | Não reage quando o local dolorido é tocado, mas reage quando é pressionado gentilmente | 1 |
|  | Reage quando o local dolorido é tocado ou pressionado | 2 |
|  | Não permite palpação | 3 |

**SPANISH - ESPAÑOL**

**Versión corta de la escala de dolor en felinos de la Unesp-Botucatu - UFEPS-SF**

([www.animalpain.org](http://www.animalpain.org))

| **ITEM** | **Descripción** | **Puntuación** |
| --- | --- | --- |
| **Evalúe la postura del gato en la jaula durante 2 minutos** | | |
| **1** | Muestra un comportamiento normal, se mueve con normalidad y/o esta relajado. | 0 |
|  | Muestra un comportamiento normal pero esta tenso, apenas se mueve, no se mueve en absoluto o no quiere moverse. | 1 |
|  | Postura encorvada y/o en decúbito dorsolateral. | 2 |
|  | Cambia de postura frecuentemente o está inquieto. | 3 |
| **2** | El gato contrae y extiende los miembros posteriores y/o contrae los músculos abdominales (del flanco). | Marque donde corresponda |
|  | Los ojos del gato están parcialmente cerrados (ignorar este punto si la evaluación se realiza durante la primera hora luego de finalizada la anestesia). |  |
|  | El gato se lame y/o muerde la zona dolorosa. |  |
|  | El gato mueve la cola enérgicamente. |  |
|  | *Todos los comportamientos anteriores están ausentes.* | 0 |
|  | *Presencia de uno de los comportamientos descriptos anteriormente.* | 1 |
|  | *Presencia de dos de los comportamientos descriptos anteriormente.* | 2 |
|  | *Presencia de tres o todos los comportamientos descriptos anteriormente.* | 3 |
|  | | |
| **Valoración del confort, actividad, actitud del gato luego de abrir la jaula y la atención prestada al observador y/o alrededores.** | | |
| **3** | Cómodo e interesado. | 0 |
|  | Quieto y con disminución del interés por interactuar. | 1 |
|  | Quieto y sin interés. El gato puede estar mirando hacia la parte trasera de la jaula. | 2 |
|  | Incómodo, inquieto, con disminución de la atención o sin interés en el entorno. Puede estar mirando hacia la parte trasera de la jaula. | 3 |
|  | | |
| **Valoración de la reacción a la palpación seguido de presión suave sobre la zona dolorosa.** | | |
| **4** | No reacciona | 0 |
|  | No reacciona cuando se palpa la zona dolorosa pero sí reacciona cuando se presiona ligeramente. | 1 |
|  | Reacciona cuando se palpa y se presiona la zona dolorosa. | 2 |
|  | No permite palpación. | 3 |
