# Supplementary material for: Multilingual validation of the short form of the Unesp-Botucatu Feline Pain Scale (UFEPS-SF)
Source: PeerJ. 2022 Mar 23;10:e13134. doi: 10.7717/peerj.13134 (PMC8957279; doi:10.7717/peerj.13134)
Supplement: Table S2 — UFEPS-SF—Unesp-Botucatu Feline Pain Scale–Short form; CI—confidence interval. ICC—intraclass correlation coefficient. Interpretation of reliability—very good 0.81–1.0; good 0.61–0.80; moderate 0.41–0.60; reasonable 0.21–0.4; poor < 0.2 (Altman, 1991; Streiner, Norman & Cairney, 2015). [file peerj-10-13134-s002.docx]

**Supplemental Table S2: Intra-observer reliability of the UFEPS-SF, unidimensional scales and rescue analgesia indication in the perioperative period of cats submitted to ovariohysterectomy (n = 30).**

| **Scale** | **Chinese** | **English** | **French** | **German** | **Italian** | | | **Japanese** | | | **Portuguese** | | | **Spanish** | | |  |
| --- | --- | --- | --- | --- | --- | --- | --- | --- | --- | --- | --- | --- | --- | --- | --- | --- | --- |
|  | **Kappa (CI)** | **Kappa (CI)** | **Kappa (CI)** | **Kappa (CI)** | **Kappa (CI)** | | | **Kappa (CI)** | | | **Kappa (CI)** | | | **Kappa (CI)** | | |  |
| **Rescue  analgesia** | 0.91 (0.84-0.99) | 0.96 (0.90-1.00) | 0.96 (0.90-1.00) | 0.95 (0.89-1.00) | 0.91 (0.83-1.00) | | | 0.98 (0.93-1.00) | | | 0.96 (0.90-1.00) | | | 0.84 (0.74-0.95) | | |  |
| **Numeric  rate** | 0.95 (0.95-0.95) | 0.97 (0.97-0.97) | 0.93 (0.93-0.93) | 0.98 (0.98-0.98) | 0.92 (0.92-0.92) | | | 0.95 (0.95-0.95) | | | 0.98 (0.98-0.98) | | | 0.95 (0.95-0.95) | | |  |
| **Simple Descriptive** | 0.90 (0.90-0.90) | 0.95 (0.95-0.95) | 0.90 (0.90-0.90) | 0.95 (0.95-0.95) | 0.87 (0.87-0.87) | | | 0.91 (0.91-0.91) | | | 0.96 (0.96-0.96) | | | 0.95 (0.95-0.95) | | |  |
| **Item 1 (posture)** | 0.84 (0.84-0.84) | 0.94 (0.94-0.94) | 0.91 (0.91-0.91) | 0.99 (0.99-0.99) | 0.90 (0.90-0.90) | | | 0.95 (0.95-0.95) | | | 0.95 (0.95-0.95) | | | 0.93 (0.93-0.93) | | |  |
|  |  |  |  |  |  |  |  |  |  |  |  |  |  |  |  |  | |
| **Item 2 (miscellaneous)** | 0.96 (0.96-0.96) | 0.97 (0.97-0.97) | 0.95 (0.95-0.95) | 0.97 (0.97-0.97) | 0.92 (0.92-0.92) | | | 0.89 (0.89-0.89) | | | 0.97 (0.97-0.97) | | | 0.96 (0.96-0.96) | | |  |
| **Item 3 (attitude)** | 0.82 (0.82-0.82) | 0.93 (0.93-0.93) | 0.79 (0.79-0.79) | 0.98 (0.98-0.98) | 0.82 (0.82-0.82) | | | 0.82 (0.82-0.82) | | | 0.93 (0.93-0.93) | | | 0.89 (0.89-0.89) | | |  |
| **Item 4 (reaction)** | 0.94 (0.94-0.94) | 0.94 (0.94-0.94) | 0.78 (0.78-0.78) | 0.97 (0.97-0.97) | 0.91 (0.91-0.91) | | | 0.87 (0.87-0.87) | | | 0.94 (0.94-0.94) | | | 0.91 (0.91-0.91) | | |  |
|  | **ICC (CI)** | **ICC (CI)** | **ICC (CI)** | **ICC (CI)** | **ICC (CI)** | | | **ICC (CI)** | | | **ICC (CI)** | | | **ICC (CI)** | | |  |
| **Visual  analogue** | 0.95 (0.93-0.96) | 0.95 (0.93-0.97) | 0.88 (0.83-0.92) | 0.98 (0.98-0.99) | 0.90 (0.86-0.93) | | | 0.94 (0.92-0.96) | | | 0.98 (0.97-0.99 | | | 0.95 (0.93-0.97) | | |  |
| **UFEPS-SF** | 0.97 (0.95-0.98) | 0.98 (0.97-0.99) | 0.94 (0.91-0.97) | 0.99 (0.99-1.00) | 0.92 (0.89-0.94) | | | 0.95 (0.93-0.97) | | | 0.98 (0.97-0.99) | | | 0.98 (0.97-0.98) | | |  |

UFEPS-SF - Unesp-Botucatu Feline Pain Scale – Short form; CI – confidence interval. ICC - intraclass correlation coefficient. Interpretation of reliability - very good 0.81 - 1.0; good 0.61 - 0.80; moderate 0.41 - 0.60; reasonable 0.21 - 0.4; poor < 0.2 (*Altman, 1991; Streiner, Norman & Cairney, 2015*).
