# Supplementary material for: Multilingual validation of the short form of the Unesp-Botucatu Feline Pain Scale (UFEPS-SF)
Source: PeerJ. 2022 Mar 23;10:e13134. doi: 10.7717/peerj.13134 (PMC8957279; doi:10.7717/peerj.13134)
Supplement: Table S3 — UFEPS-SF—Unesp-Botucatu Feline Pain Scale–Short form; CMPS-Feline—Glasgow Composite Multidimensional Pain Scale (Reid et al., 2017); ICC—intraclass correlation coefficient. Interpretation of reliability—very good 0.81–1.0; good 0.61–0.80; moderate 0.41–0.60; reasonable 0.21–0.4; poor < 0.2 (Altman, 1991; Streiner, Norman & Cairney, 2015). [file peerj-10-13134-s003.docx]

**Supplemental Table S3. Interobserver reliability of all evaluators for the UFEPS-SF, CMPS-Feline and unidimensional scales in the perioperative period of cats submitted to ovariohysterectomy (n = 30).**

| **Scale** | **Weighed Kappa** | **Confidence interval** |
| --- | --- | --- |
| **Rescue analgesia** | 0.66 – 0.98 | 0.56 – 1 |
| **Numerical rating scale** | 0.80 - 0.96 | 0.8 - 0.96 |
| **Simple descriptive scale** | 0.77 - 0.96 | 0.76 - 0.96 |
| **Item 1 (posture)** | 0.82 - 0.94 | 0.81 - 0.94 |
| **Item 2 (miscellaneous)** | 0.86 - 0.94 | 0.82 - 0.95 |
| **Item 3 (attitude)** | 0.70 - 0.9 | 0.70 - 0.9 |
| **Item 4 (reaction to palpation)** | 0.75 - 0.94 | 0.75 - 0.94 |
|  | **Intraclass correlation**  **coefficient** | **Confidence interval** |
| **Visual analog scale** | 0.79 - 0.95 | 0.77 - 0.96 |
| **UFEPS-SF** | 0.84 - 0.97 | 0.80 - 0.98 |
| **CMPS-Feline** | 0.78 - 0.96 | 0.79 - 0.97 |

UFEPS-SF - Unesp-Botucatu Feline Pain Scale – Short form; CMPS-Feline - Glasgow Composite Multidimensional Pain Scale (*Reid et al., 2017*); ICC - intraclass correlation coefficient. Interpretation of reliability - very good 0.81 - 1.0; good 0.61 - 0.80; moderate 0.41 - 0.60; reasonable 0.21 - 0.4; poor < 0.2 (*Altman, 1991; Streiner, Norman & Cairney, 2015*).
