# Supplementary material for: Multilingual validation of the short form of the Unesp-Botucatu Feline Pain Scale (UFEPS-SF)
Source: PeerJ. 2022 Mar 23;10:e13134. doi: 10.7717/peerj.13134 (PMC8957279; doi:10.7717/peerj.13134)
Supplement: Table S4 — UFEPS-SF—Unesp-Botucatu Feline Pain Scale–Short form. Items with a load value ≥ 0.50 or ≤−0.50 (in bold) were considered with representative dimension (eigenvalue > 1 and variance > 20%) (Kaiser, 1958). [file peerj-10-13134-s004.docx]

**Supplemental Table S4. Load values, eigenvalues and variance of the UFEPS-SF items based on principal components analysis** **(n = 30 cats).**

|  | **Load values** | |
| --- | --- | --- |
| Items | **Dimension 1** | **Dimension 2** |
| **1 - posture** | **0.95** | 0.17 |
| **2 - miscellaneous** | **0.95** | 0.14 |
| **3 - attitude** | **0.94** | 0.18 |
| **4 - reaction to palpation** | **0.83** | **-0.56** |
| **Eigenvalue** | 3.38 | 0.39 |
| **Variance** | 84.34 | 9.83 |

UFEPS-SF - Unesp-Botucatu Feline Pain Scale – Short form. Items with a load value ≥ 0.50 or ≤ -0.50 (in bold) were considered with representative dimension (eigenvalue > 1 and variance > 20%) (*Kaiser, 1958*).
