# Supplementary material for: Multilingual validation of the short form of the Unesp-Botucatu Feline Pain Scale (UFEPS-SF)
Source: PeerJ. 2022 Mar 23;10:e13134. doi: 10.7717/peerj.13134 (PMC8957279; doi:10.7717/peerj.13134)
Supplement: Table S5 — UFEPS—Unesp-Botucatu Feline Pain Scale (Brondani et al., 2013b); UFEPS-SF—Unesp-Botucatu Feline Pain Scale–Short form; CMPS-Feline—Glasgow Composite Multidimensional Pain Scale (Reid et al., 2017). Interpretation of Spearman’s correlation coefficient: <0.19: very weak; 0.2–0.39: weak; 0.4–0.59: moderate; 0.6–0.79: strong, 0.8–1: very strong (Evans, 1996). P values were less than < 0.01−27 in all cases. [file peerj-10-13134-s005.docx]

**Supplemental Table S5. Criterion validity based on the Spearman correlation between the UFEPS-SF versus UFEPS, CMPS-Feline and unidimensional scales to assess perioperative pain in cats (n = 30).**

| **Scales** | **UFEPS-SF** | **CMPS-Feline** |
| --- | --- | --- |
| **Numerical rating scale** | 0.93 | 0.91 |
| **Simple descriptive scale** | 0.92 | 0.91 |
| **Visual analog scale** | 0.90 | 0.89 |
| **CMPS-Feline** | 0.92 |  |
| **UFEPS** | 0.91 | 0.81 |

UFEPS - Unesp-Botucatu Feline Pain Scale (*Brondani et al., 2013c*); UFEPS-SF - Unesp-Botucatu Feline Pain Scale – Short form; CMPS-Feline - Glasgow Composite Multidimensional Pain Scale (*Reid et al., 2017*). Interpretation of Spearman’s correlation coefficient: < 0.19: very weak; 0.2–0.39: weak; 0.4–0.59: moderate; 0.6–0.79: strong, 0.8–1: very strong (*Evans, 1996*). P values were less than < 0.01^-27^ in all cases.
